# Toxic Effects on Bioaccumulation, Hematological Parameters, Oxidative Stress, Immune Responses and Tissue Structure in Fish Exposed to Ammonia Nitrogen: A Review

**DOI:** 10.3390/ani11113304

**Published:** 2021-11-19

**Authors:** Zhenkun Xu, Jie Cao, Xiaoming Qin, Weiqiang Qiu, Jun Mei, Jing Xie

**Affiliations:** 1 College of Food Science and Technology, Shanghai Ocean University, Shanghai 201306, China; m200300892@st.shou.edu.cn (Z.X.); m190300743@st.shou.edu.cn (J.C.); wqqiu@shou.edu.cn (W.Q.); 2 National Experimental Teaching Demonstration Center for Food Science and Engineering, Shanghai Ocean University, Shanghai 201306, China; 3 Shanghai Engineering Research Center of Aquatic Product Processing and Preservation, Shanghai 201306, China; 4 Shanghai Professional Technology Service Platform on Cold Chain Equipment Performance and Energy Saving Evaluation, Shanghai 201306, China; 5 College of Food Science and Technology, Guangdong Ocean University, Zhanjiang 524088, China; qinxm@gdou.edu.cn

**Keywords:** ammonia nitrogen, fish, oxidative stress, neurotoxicity, immune response

## Abstract

**Simple Summary:**

Ammonia nitrogen is a common environmental limiting factor in aquaculture, which can accumulate rapidly in water and reach toxic concentrations. In most aquatic environments, fish are vulnerable to the toxic effects of high levels of ammonia nitrogen exposure. It has been found that the toxic effects of ammonia nitrogen on fish are multi-mechanistic. Therefore, the purpose of this review is to explore the various toxic effects of ammonia nitrogen on fish, including oxidative stress, neurotoxicity, tissue damage and immune response.

**Abstract:**

Ammonia nitrogen is the major oxygen-consuming pollutant in aquatic environments. Exposure to ammonia nitrogen in the aquatic environment can lead to bioaccumulation in fish, and the ammonia nitrogen concentration is the main determinant of accumulation. In most aquatic environments, fish are at the top of the food chain and are most vulnerable to the toxic effects of high levels of ammonia nitrogen exposure. In fish exposed to toxicants, ammonia-induced toxicity is mainly caused by bioaccumulation in certain tissues. Ammonia nitrogen absorbed in the fish enters the circulatory system and affects hematological properties. Ammonia nitrogen also breaks balance in antioxidant capacity and causes oxidative damage. In addition, ammonia nitrogen affects the immune response and causes neurotoxicity because of the physical and chemical toxicity. Thence, the purpose of this review was to investigate various toxic effects of ammonia nitrogen, including oxidative stress, neurotoxicity and immune response.

## 1. Introduction

Ammonia nitrogen is the final product of protein catabolism and metabolism. In farming water, ammonia nitrogen is mainly derived from the decomposition process of organic matter. It is the most common environmental limiting factor in aquaculture [1]. The ammonia concentration in naturally occurring water is approximately 0–0.2 mg/L, which is at a low level. However, with the development of culturing systems, the excessive concentration of ammonia nitrogen in farmed water has become a common environmental problem. Ammonia has the greatest impact on the physiological function of aquatic organisms. Ammonia can accumulate quickly and reach toxic concentrations [2]. There are two main reasons for the growth of the mass concentration of ammonia nitrogen in farming water: one is the decomposition of organic matter containing nitrogen such as feed residues and aquatic animal excreta [3]. High-density farming increases the burden of these substances, resulting in the accumulation of ammonia nitrogen. The second is industrial wastewater, domestic sewage discharge, etc. In various regions of fertilization of pesticides agriculture, the massive use of nitrogen fertilizers, mainly ammonium chloride and urea, entering the farming water with the washout of rainwater and surface runoff [4].

Ammonia nitrogen is the sum of ammonia present in water in two forms of ionized (NH_4_^+^) and non-ionic ammonia (NH_3_), which is also called total ammonia (TAN) [5]. The equilibrium reaction equation of both is

(1) $${\mathrm{NH}}_{4^{+}}+{\mathrm{OH}}^{-}\;⇄\;{\mathrm{NH}}_{3}\cdot H_{2}O\;⇄\;{\mathrm{NH}}_{3}\;+H^{+}$$

Ammonia nitrogen is the main product of fish metabolism, and most fish are sensitive to ammonia [6]. The increase of non-ionic ammonia in the water environment will inhibit the excretion of ammonia nitrogen in the fish and increase the concentration of ammonia in their blood and tissues, making the blood less capable of carrying oxygen and disrupting normal metabolism. Non-ionic ammonia is the main toxic form of physiological stress in fish and is about 300 to 400 times more toxic than ionized ammonia [4]. Non-ionic ammonia has good lipid solubility and carries no electric charge, making it more easily fuse with the phospholipid bilayer on the cell membrane, thus diffusing through the cell membrane to the hemolymph and increasing the concentration of ammonia nitrogen in fish, producing toxic effects [7]. Furthermore, non-ionic ammonia has good lipid solubility. It can easily diffuse into fish through cell membranes, causing damage to important organs, including gill tissue, resulting in respiratory difficulties and reduced feeding rate of fish, and inhibiting their growth and development [8]. The non-ionic ammonia which enters the fish directly affects the metabolism of enzymes, causing disorders of enzyme metabolism and reducing immunity, stimulating a series of toxic reactions in fish, such as excitement and convulsions until they die of exhaustion [9,10].

**Figure 1:**
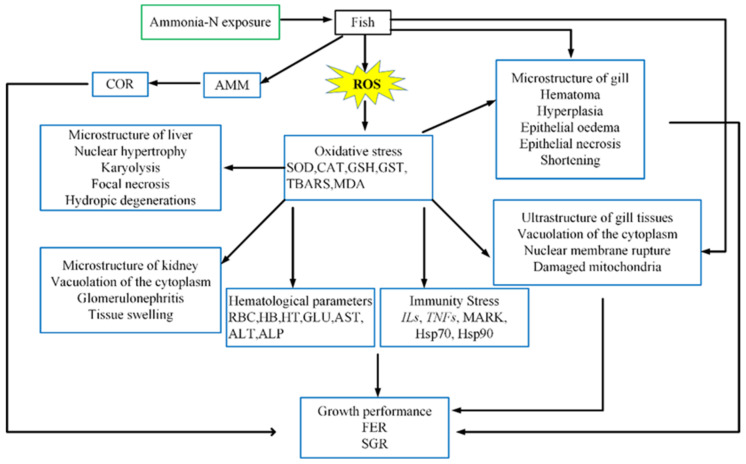
Toxicological characteristics and physiological response process for fish exposed to ammonia [11]. AMM: ammonia; COR: cortisol; SOD: superoxide dismutase; CAT: catalase; GSH: glutathione; GST: Glutathione S-transferase; TBARS: Thiobarbituric reactive substances; MDA: malon dialdehyde; RBC: red blood cell count; Hb: hemoglobin; Ht: hematocrit; GLU: glucose; AST: aspartate transaminase; ALT: alanine transaminase; ALP: alkaline phosphatase; *Ils*: interleukins; *TNFs*: tumor necrosis factors; FER: feed efficiency ratio; SGR: specific growth—Reprinted with permission from ref. [11]. Copyright 2019 Elsevier.

We comprehensively analyzed the main toxicological characteristics and physiological reaction processes of fish after exposure to ammonia (Figure 1). The toxicity of ammonia nitrogen is manifold, and therefore it is necessary to conduct an overall research and analysis to confirm the adverse effects of ammonia nitrogen on fish. Thus, the aim of this review was to summarize changes in ammonia accumulation patterns, oxidative stress, neurotoxicity and changes in immunological reactions in fish following exposure to ammonia nitrogen.

## 2. Bioaccumulation

The toxicity of ammonia to fish mostly results from bioaccumulation as well as excretion, metabolism and detoxification mechanisms caused by ammonia nitrogen uptake [12]. In water, ammonia is present as non-ionic ammonia (NH_3_) and ionized ammonium (NH_4_^+^), the latter accounting for a large proportion at normal water pH [3]. Most biofilms are not permeable to ammonium ions but are permeable to ammonia. Thus, the ammonia toxicity is owing to its non-ionic form (NH_3_), which can easily spread through gill membranes [13]. Furthermore, provided there is an outward gradient, the ammonia can be discharged into water as NH_3_ through gills, and this process is aided by Rhesus (Rh) glycoproteins [14]. However, under high ambient ammonia (HEA), the external ammonia flux through gills is decreased and the inverse internal flux of ammonia occurs. Therefore, ammonia levels in blood and tissues increased and acute and chronic toxic reactions were observed in fish [15,16].

Ammonia toxicity to fish has been demonstrated to follow a multifactorial pathogenesis. It is generally considered that NH_3_ enters the organism through the gills, epidermis, and intestinal mucosa of fish, which increases the blood pH and reduces oxygen carrying capacity of blood. Long-term exposure to ammonia also damages gill tissue, leading to gill tissue congestion, gill lamellae bending and adhesion, affecting gill gas exchange, inhibiting the respiratory function of the organism, leading to fish hypoxia and even death [7]. After ammonia diffuses into the tissues, the ammonia level increases [17], damaging the liver and kidney system, resulting in congestion, edema, liver coma and even death [18].

Ammonia can also damage the central nervous system of fish. Initially, researchers believed that mechanisms of ammonia poisoning of fish were similar to those of hepatic encephalopathy in mammals [19]. This is because high levels of non-ionic ammonia (NH_3_) in the brain synthesize high amounts of glutamine (Gln) catalyzed by glutamine synthetase, swelling neuroglia and activating the N-methyl-D-aspartate (NMDA) receptor [20]. Under normal conditions, the organism produces Gln mainly from glutamate (Glu) and NH_4_^+^ to remove excess NH_4_^+^ from the brain. Therefore, increased concentration of NH_4_^+^ in the brain is often accompanied by an increase in the level of Gln. While the excessive increase in Gln intracellularly leads to astrocyte swelling [21], triggering brain edema and promoting the release of Glu into the intercellular, causing intracranial hypertension and death [22]. In addition, NH_4_^+^ also causes depolarization of the neuronal surface. Both Glu and depolarized neurons accumulated intercellularly can activate NMDA receptors located on the neuronal surface. Excessive activation of this receptor promotes NO synthesis and activates Na^+^/K^+^-ATPase [23,24]. Activation of Na^+^/K^+^-ATPase accelerates energy depletion in the brain. The excessive amount of NO not only increased the oxidative stress in the organism, but also tends to produce extremely toxic hydrogen peroxide, which impairs the mitochondrial respiration, initiates ATP exhaustion and results in cell death [25,26]. This assumption has been accepted by most scholars for a long time. In the early 21st century, several studies confirmed that some fish, such as *Cyprinus* and mudskipper, accumulate glutamine after ammonia stress at concentrations much greater than those that constitute lethal concentrations in mammals [27,28]. The histological data suggested that the skull of fish has more cranial space than mammals and they can bear more cranial pressure [11]. Therefore, fish may have different defense mechanisms to ammonia toxicity than mammals.

Nevertheless, physical stress responses (or pathological processes) could be triggered by the production of metabolites in adaptive regulation, for example, the oxidative damage and inflammatory hyperinflammation, which could be the real reason for fish death [29]. Likewise, Hegazi et al. indicated that ammonia nitrogen stress triggers the formation of reactive oxygen species, which are also mediators of ammonia toxicity [30]. In recent years, with the continuous innovation of research methods in aquatic animals, new evidence suggests that ammonia toxicity in fish is associated with the over inflammatory response of the organism [31,32]. The exact mechanisms of ammonia toxicity to fish need to be further examined.

## 3. Hematological Parameters

The inflow of toxic materials to the water environment affects water parameters and leads to alterations in the fish hematological profile [33,34]. Toxic exposure can adversely affect the blood oxygen carrying capacity and the blood electrolyte balance, especially ammonia exposure can induce the accumulation of ammonia in fish circulatory system. As ammonia has high affinity for blood hemoglobin, it displaces oxygen and influences some hematological properties [35]. Hematological properties are significant indicators to assess fish health status after exposed to different environmental stresses and chemical toxicity [36,37,38,39]. Ammonia enters the fish circulatory system and causes metabolic disturbances and fatal responses, such as oxidative stress, immune response and genetic expression [40]. Exposure to ammonia also affects the fish circulatory system, particularly hematological indexes such as red blood cell count (RBC), hematocrit (Ht) and hemoglobin (Hb) [32]. In general, exposure to toxic substances decreases hematological properties like RBC, Ht and Hb owing to the hemolysis and red blood cells destruction and may cause anemia [41]. Praveena et al. suggested that the decrease in RBC and the concentration of hemoglobin resulting from toxicity exposure might be attributed to the destructive effects of toxicity, but the decrease in the concentration of hemoglobin resulted in a potential impairment of tissue function because of the inadequate oxygen supply to the tissues [42]. Gao et al. observed significant declines in Hb content, Ht and blood RBC counts after exposure to high concentrations of ammonia in *Takifugu rubripes*, suggesting that the fish was anemic by exposure to ammonia [40]. Hoseini et al. revealed that the increase in radicals exposed to ammonia could lead to attack on RBCs, leading to their destruction [43]. Researches have revealed that the ammonia toxicity induces the inhibition of hematopoiesis by destroying the production sites of red blood cells [44]. The onset of anemia symptoms may be due to destruction of red blood cells or injury to hematopoietic tissues after exposure to ammonia. Serum proteins are considered to be reliable indicators of fish immune status and health [45]. David et al. attributed the decreased protein content in the toxically exposed fish to the disruption or collapse of cell functions and the concomitant impairment of protein synthetic mechanisms [46]. Asthana et al. reported that high concentration of ammonia resulted in the deamination of proteins and increased the degradation of proteins [47]. Ammonia exposure induced a reduction in hematological properties like RBC, Ht and Hb in fish, which is considered to account for the stress-induced decline in Hb content and Hb synthesis rate. Thus, it may exert a toxic effect by inducing disturbances in tissue oxygenation.

Ammonia absorbed in fish diffuses through cell membranes into the blood system and causes accumulation. Therefore, hematological parameters usually act as sensitive indicators to evaluate the toxicity of ammonia to fish [48]. The effects of ammonia on fish hematological parameters are shown in Table 1, regarding the route of exposure (freshwater, seawater, waterborne exposure). The decrease in hematological properties induced by ammonia exposure is manifested by disruption of RBC and alteration of the small or large red blood cell anemic state. Das et al. reported changes in blood characteristics (such as RBCs and hemoglobin) of *Cirrhinus mrigala* after exposure to ammonia [49]. This phenomenon causes tissue damage due to ammonia toxicity and hemodilution after hemolysis. Iheanacho et al. showed that changes in RBC content, hematocrit values and hemoglobin concentration reflected the fish defensive mechanisms against stress induced by exposure to environmental toxicity [50]. Kim et al. revealed that the ammonia exposure greatly decreased the levels of hematocrit and hemoglobin in juvenile hybrid grouper [48]. These authors considered that fish tissues might be under hypoxic conditions because of ammonia exposure and may result in the inhibition and depletion of hematopoietic potential under that condition.

The balance of glucose levels is maintained by balancing the production of glucose and the storage of glucose as glycogen [51]. Glucose metabolism meets the energy requirements of the organs and tissues, which can mediate the ammonia response. Zhao et al. reported a significant increase in glucose in juvenile yellow catfish, *Pelteobagrus fulvidraco*, exposed to ammonia [52]. Long-term exposure to ammonia results in a dramatic elevation of blood glucose in *Litopenaeus vannamei* owing to the impaired glucose metabolism in the liver [53]. Lower glucose levels were critical for reducing tissue injuries and maintaining low levels of gene expression of pro-inflammatory cytokines in stressful conditions [32]. Changes in glucose levels in fish caused by ammonia exposure were attributed to stress responses or disturbances in homeostasis.

Enzyme plasma components including aspartate transaminase (AST), alanine transaminase (ALT) and alkaline phosphatase (ALP) are recognized as credible and sensitive biological indicators for evaluating damages to the liver and other fish organs following environmental stress [54]. Plasma ALT and AST levels play important roles in indicating hepatopancreatic functions and injuries, and can be used as sensitive indicators of hepatocyte integrity [55]. Zhao et al. revealed that AST and ALT levels were significantly increased in juvenile yellow catfish, *Pelteobagrus fulvidraco*, exposed to ammonia, which may be due to damage to cell membranes and liver [52]. Hoseini et al. recorded significant increases in ALT, AST and ALP of *Cyprinus carpio* following ammonia exposure, which may be due to damage to cell membranes [43]. Zhang et al. reported that ALP levels were significantly increased in *Megalobrama amblycephala* exposed to ammonia [11]. ALP is an important indicator reflecting liver damage, so the upward trend in ALP in *Megalobrama amblycephala* is thought to be attributed to liver damage and stress caused by ammonia exposure. Peyghan et al. reported that ammonia exposure induced a remarkable increase in ALP in *Cyprinus carpio*, indicating that hematological parameters were affected [56].

Ammonia enters the circulation and disrupts blood proteins participated in lipid metabolism, immune defense, blood coagulation and molecular transport, when fish are exposed to ammonia [11,13]. Specifically, ammonia particles may affect various blood physiologies through chemical and physical interactions in the blood. Various studies have confirmed changes in various hematological properties following ammonia exposure, and hematological parameters may be a reliable indicator for assessing fish toxicity.

## 4. Oxidative Stress

**Figure 2:**
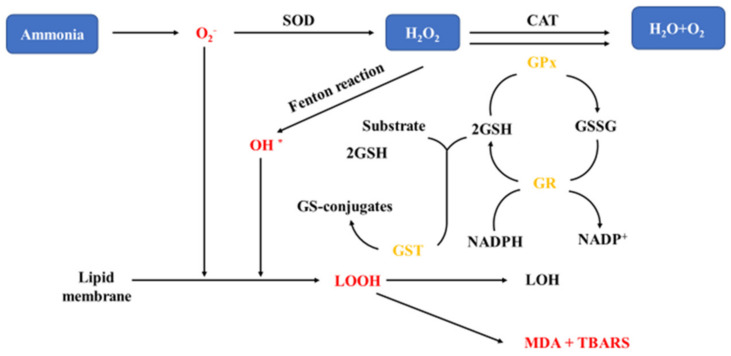
Oxidative stress mechanisms in fish exposed to ammonia [81]. H_2_O_2_: hydrogen peroxide; SOD: superoxide dismutase; CAT: catalase; GSH: glutathione; GST: Glutathione S-transferase; TBARS: Thiobarbituric reactive substances; MDA: malon dialdehyde; GPx: glutathione peroxide; GR: glutathione reductase; GSSG: glutathione; NADPH: nicotinamide adenine dinucleotide phosphate—Reprinted with permission from ref. [81]. Copyright 2021 Elsevier.

Oxidative stress is one of the toxicity mechanisms of ammonia nitrogen stress in aquatic animals [7]. It has been shown that the increase in the concentration of ammonia nitrogen in aquaculture water can result in the production of reactive oxygen species (ROS) in aquatic animals [60]. ROS combines with unsaturated fatty acids and cholesterol on the cell membrane to produce lipid peroxidation, which leads to reduced mobility and greater cell membrane permeability. Disturbance of the distribution of proteins across the cell membrane leads to cell membrane dysfunction and apoptosis [61,62]. In order to counteract antioxidant stress and maintain the balance of the redox state of cells, antioxidant defense systems have evolved to function at different levels to avoid or repair this damage [31]. The mechanism of oxidative stress in fish exposed to ammonia is shown in Figure 2. Studies have reported that the activities of antioxidant enzymes can be elicited in low concentrations of pollutants and disrupted in high concentrations [63,64]. When physiological antioxidant system is unable to counteract the increased levels of stress-generated ROS, cellular oxidative stress occurs [65].

Table 2 shows the responses of antioxidant enzymes in fish exposed to ammonia. The oxidative stress in fish exposed to ammonia stress is indicated by changes in the production of ROS in fish. One of the main defense strategies to reduce ROS production is by raising the activity of antioxidant enzymes, including superoxide dismutase (SOD), catalase (CAT), and glutathione (GSH) [35,65,66,67,68,69]. Antioxidant enzymes are widely present in tissues and are most abundant in hepatocytes [70,71]. Under stress, fish can protect the structure and function of cell membranes from peroxides by converting O_2_^−^ to H_2_O_2_ through SOD, GSH and CAT and by breaking down cytotoxic H_2_O_2_ into oxygen and water [72].

SOD is first defense against antioxidant stress [73]. As a primary defense mechanism against antioxidant stress, SOD transforms superoxide radicals into hydrogen peroxide (H_2_O_2_). SOD activity in ammonia-exposed fish is usually increased due to defense mechanisms against ROS production [74]. Changes in SOD concentration appear to be associated with differences ammonia tolerance of fish. The SOD activity of rainbow trout, carp, goldfish and *Dicentrarchus labrax* did not change significantly under ammonia stress [75,76]. SOD activity decreased significantly in *Carassius auratus* and *Litopenaeus vannamei* [9,77]. While in *Takifugu obscurus* and *Pelteobagrus vachelli*, SOD activity increased significantly [78,79]. However, excessive accumulation of free radicals inhibits antioxidant enzyme capacity to scavenge ROS, and a significant downward trend in SOD activity is observed after a remarkable increase in SOD activity. Kim et al. found that SOD activity in the liver and gills significantly increased when juvenile hybrid grouper exposure to ammonia. However, SOD activity in the gills had a downward trend when fish was subjected to high levels of ammonia. [48]. Sun et al. revealed that when bighead carps were exposed to high concentrations of ammonia, the SOD activity of bighead carp, *Hypophthalmythys nobilis*, larvae first increased and then decreased [80]. The SOD activity may be stimulated in response to excessive ROS production, but then SOD activity declines because they are unable to perform under higher ammonia concentrations.

Ammonia exposure also leads to reduction in antioxidant enzymes as the energy expended in response to antioxidant stress. CAT is the major antioxidant enzyme to eliminate H_2_O_2_, a by-product of SOD, thus reducing its toxic effects [35,69,82]. Xue et al. showed that ammonia exposure can depress normal ROS-mediated oxidative processes and reported a reduction of CAT activity in *Cyprinus carpio* following ammonia exposure [83]. Zhang et al. reported that ammonia exposure decreases SOD and CAT in the digestive gland of *Corbicula fluminea* after an initial increase. This phenomenon is thought to be caused by the inhibition of antioxidant enzymes, as the ROS generated in the tissues are not cleared immediately. The activity of CAT also declined with increasing the concentration of ammonia, which indicated the oxidative damage and stress [84]. In addition, Wang et al. concluded that the short time increase in antioxidant enzyme activity with treatment was not sufficient to fully counteract stress-induced cellular damage [85]. In normal conditions, ROS are quickly removed by the antioxidant defense system. However, stimulated by large amounts of ammonia, excess ROS were produced, disrupting the cell membrane, forming lipid peroxides and oxidized proteins, and the balance between oxidants and antioxidants was disrupted. The body’s detoxification function was severely inhibited.

Glutathione S-transferase (GST) functions in the second stage of the fish detoxification metabolism by conjugating to xenobiotics and clearing them from the cells, and the activity of GST is usually triggered in fish exposed to environmental toxins [69]. Thus, GST plays a key role in homeostasis and foreign body dissociation, protecting tissues from oxidative stress of toxicant exposure [86]. Many scholars have reported that ammonia exposure affects GST activity in fish through the induction of oxidative stress [10,75,87]. Maltez et al. suggested that GST activity in Brazilian flounder (*Paralichthys orbignyanus*) increased as a result of ammonia exposure and that ROS increased by ammonia exposure stimulated antioxidant defense [88]. Kim et al. reported that GST activity in juvenile hybrid grouper initially increased significantly and tended to decrease with higher ammonia exposure concentration and time [48]. Li et al. also reported a significant upward trend and subsequent downward trend in GST activity in carp following exposure to acute ammonia gas [10]. The decrease in GST after the initial increase may be caused by excessive ROS production, which is consistent with the changes observed in SOD activity.

Thiobarbituric reactive substances (TBARS) was used as a measure of lipid peroxidation [89]. It is well known that oxidative stress is caused by lipid peroxidation, leading to loss of cellular function [90]. TBARS is a sensitive indicator for estimating lipid peroxidation as its products are produced by peroxidation of membrane lipids [91]. Li et al. reported a gradual rise in brain TBARS levels in *Pelteobagrus fulvidraco* exposed to high ammonia. They suggested that elevated brain TBARS levels could be an essential factor in the pathogenesis of ammonia toxicity in fish exposed to high concentrations of ammonia [92]. Almroth et al. suggested that lipid peroxidation in fish occurs during oxidative stress from environmental toxicants [93]. Aldehydes and ketones scan to crosslink with nucleophilic moieties of proteins, nucleic acids and aminophospholipids, and greater levels of TBARS result in increased cytotoxicity and accelerated cell and tissue damage [94]. In particular, as fish contain many highly unsaturated fatty acids (HUFA), TBARS can be used as a biomarker of oxidative stress [95].

Excessive ROS production induces oxidative damage when fish were exposed to toxic substances, such as ammonia nitrogen. Excess ROS may damage cell membranes, form lipid peroxides and oxidize proteins [33]. Usually, MDA is used as a biomarker of lipid peroxidation because it is an important product of membrane lipid peroxidation following free radical attack on biological membranes [96,97]. The changes of MDA content can indirectly reflect the level of disruption of biofilm system. Li et al. found no significant changes in the MDA content of *Cyprinus carpio* L. during exposure to 10 mg/L ammonia water. However, after 48 h of exposure to 30 mg/L ammonia, MDA levels increased significantly [10]. Xue et al. found that MDA levels had an upward trend when *Cyprinus carpio* exposed to ammonia [83]. The glutathione redox system could be triggered by ammonia stress. However, the antioxidant reaction is not sufficient to prevent oxidative damage caused by increased ammonia concentration. Ammonia exposure has toxic effects on fish through the induction of oxidative stress and ROS production, and antioxidant enzymes in fish such as SOD, CAT, GST, TBARS and MDA are the main indicators to reflect oxidative stress caused by ammonia.

## 5. Immune Response

When fish are exposed to ammonia it induces changes in the immune response of fish [49]. Gut damage in fish caused by ammonia stress can directly affect the immune system, resulting in intestinal inflammation and cytokine expression [99]. Ammonia exposure can induce the production of immunosuppressive factors in peripheral lymphoid tissues and release into blood; it leads to a decline in lymphocytes and phagocytes and suppression of the immune response. Das et al. reported that the total lymphocyte count (TLC) increased as a result of immune system stimulation and tissue damage in ammonia-exposed fish; however, prolonged exposure leads to a decrease in lymphocytes and white blood cells, which is due to damage to the immune system [49]. Prolonged exposure to higher concentrations of toxicants can lead to a failure in the production of this TLC, resulting in a decrease in non-specific immunity in fish [100]. Yan et al. reported that ammonia exposure affected the immune responses to immune factors such as plasma immunoglobulin M (IgM), which may be due to stimulation of the immune system in response to ammonia [12].

Ammonia is known to influence the immune response through control of cytokine expression [101]. Cytokines, including interleukins (ILs) and tumor necrosis factors (TNFs), are commonly used as markers to determine acute macrophage-associated inflammatory responses [102]. Among these cytokines, IL-1β and IL-6 are involved in cellular inflammatory immune responses, and TNF-α is involved in inflammation, apoptosis and immune responses [103,104]. Li et al. revealed increased mRNA expression of IL-1β and TNF-α in *Pelteobagrus fulvidraco* exposed to ammonia, suggesting that ammonia triggered the cellular inflammatory response [99]. He et al. reported that the levels of IL-1, IL-6 and TNF-α were significantly increased in ammonia-exposed head kidney macrophages and that certain ammonia concentrations may be severely detrimental to immune system activity in fish [101].

Ammonia affects the immune response by triggering the activity of mitogen-activated protein kinase (MAPK). MAPK is important for transmitting various signals to nucleus, including stress response and apoptosis [105,106]. MAPK consists of three subcomponents: ERKs, JNKs and p38 [107]. In addition, ammonia stimulates the expression of Hsp70 and Hsp90 in fish [12]. Cheng et al. reported that environmental ammonia stress altered the Hsp70 and Hsp90 transcription in *Takifugu obscurus* [31]. Hsp70 is known to be involved in stress response, intracellular trafficking, anti-apoptosis, antigen processing or chaperone functions [108,109]. Hsp90 is usually present in a constitutively dimmable manner and functions critically in the control of multiple regulatory pathways, for example, stress defense, hormonal signaling, cell cycle control, cell proliferation and differentiation, and apoptosis [110].

Many studies have shown that ammonia can harm immune responses in fish, such as lymphocytes, white blood cells, inflammation and apoptosis. Ammonia acts as an immunotoxic agent not only by inhibiting the activity of various biomolecules, but also by interfering with intracellular signal transduction. Thus, changes in fish immune responses have been used as important indicators to identify the toxic effects of ammonia exposure.

## 6. Tissue Structure

Oxidative damage caused by ammonia exposure is closely related to the physiological toxicity of fish, and long-term ammonia exposure is toxic to the fish organism, leading to tissue lesions and physiological dysfunction [7,111]. Fish organs, such as gills, livers and kidneys, play important roles in defending against environmental toxicity. Gills are the first organs to be responsive to harmful environmental conditions [112]. The liver is an important organ for detoxification, and it is vulnerable affected by water pollutants such as ammonia [113]. The kidneys also serve as the main detoxification organs and are the target organs for ammonia poisoning [114,115]. Many studies have found that high concentrations of ammonia are harmful to fish health, causing immunosuppression; high mortality; and damage to gill, liver and kidney tissues in fish [7,79].

The gill is a well-known target organ in fish; it is the first organ to come into contact with pollutants in the water column and is an important endpoint for toxic effects during ammonia nitrogen stress [116]. The gill epithelium is an important site for gas exchange and ion regulation in aquatic organisms. In the present studies, the histopathological effects of acute ammonia exposure on gills were chloride cell proliferation, hyperemia, epithelial elevation, secondary laminae convolution, laminae fusion, and secondary laminae shortening [117,118]. Zhang et al. found histopathological changes in gill tissues of all *Megalobrama amblycephala* exposed to different concentrations of ammonia by pathological histological examination, which revealed cytoplasmic vacuolation, damaged mitochondria, increased heterochromatin and significant epithelial cell detachment in gill tissue [119]. Ricardo et al. found that ammonia exposed maroon clownfish gills exhibited lamellar deformation, lamellar shortening, mucosal cell hyperplasia and hypertrophy [111]. In addition, some researchers believe that the effects of ammonia on gills depend on the duration of exposure to ammonia. Rodrigues et al. recorded that a significant proliferation of chloride cells was observed in juvenile cobia (*Rachycentron canadum*) after 6 h of exposure to ammonia [120]. Chloride cell hyperplasia implies changes in the amount of Na+/K+-ATPase as a defense response in fish, which may cause impaired diffusion of oxygen in the gills and impairment of osmoregulatory function [121,122]. Zhang et al. observed that shortening of secondary lamellae in *Megalobrama amblycephala* after exposure to ammonia for 24 h, which indicated that ammonia pressure has direct, harmful and irreversible toxic effects.

The liver is an important organ for detoxification, and the toxic effects of chemicals are usually first seen in the liver. Ammonia can enter the hepatic metabolic pathway as a nutrient, but it is also an environmental pollutant that affects fish health [113]. The liver portal vein carries ammonia nitrogen as a nutrient into the hepatic metabolic pathway [123]. Due to the interruption of production capacity, ammonia nitrogen exposure can cause a significant reduction in liver glycogen cytoplasm or even the formation of vacuoles, usually with alterations including edematous degeneration and cloudy swelling, vacuolization, and localized necrosis [124,125]. Benli et al. observed cloudy swelling and hydropic degenerations on the liver in *Oreochromis niloticus* L. when they were subjected to sublethal concentrations of ammonia nitrogen [7]. Zhang et al. also observed large fields of nuclear hypertrophy in the liver, which indicated that the liver of juvenile blunt snout bream could not fully recover after 96 h post-exposure [119]. Therefore, ammonia exposure can cause structural disruptions in fish livers and make it difficult to recover.

The kidney is one of the main organs involved in detoxification and is therefore a target organ for ammonia poisoning [88]. Ammonia pressure can also cause swelling, blockage and bleeding in the renal tubules and capsule. Ravindrababu et al. observed vacuolation and degeneration of renal tubular epithelial cells in the kidneys of *Oreochromis mossambicus* after 14 days of exposure to ammonia nitrogen, and the intensity of damage to renal tubules was more clearly observed in exposed fish at 14 days than at 7 days [126]. Zhang et al. observed renal tubular dilatation and severe histopathological damage (vacuolation and degeneration of the renal tubular epithelium) in the kidneys of juvenile blunt snout bream after 24 h of exposure [119]. The kidneys of Nile tilapia (*Oreochromis niloticus* L.) exposed to ammonia nitrogen exhibited pathological features such as glomerulonephritis and congestion [7]. Many studies have reported structural tissue damage in fish caused by ammonia exposure, and indicators of tissue structure can be used as important biomarkers to assess toxicity after ammonia exposure.

## 7. Conclusions

Ammonia exposure could trigger many toxic effects to fish, affecting physiological and biochemical functions. Ammonia accumulation in fish tissues disturbs circulatory systems and various hematological parameters related to lipid metabolism, immune defense system, blood coagulation and molecular transport. Ammonia accumulation in fish induces oxidative stress through the production of a large amount ROS. Ammonia accumulation leads to the activation of antioxidant enzymes such as SOD, CAT and TBARS are activated in fish to alleviate the adverse of oxidative stress. Ammonia exposure also causes tissue damage including fish gills, livers and kidneys through oxidative damage and physiological toxicity. Immune responses can also be modified by the toxic effect of ammonia exposure. Ammonia accumulation can lead to immune responses in fish, such as lymphocytes, white blood cells, inflammation and apoptosis. In summary, fish exposed to ammonia can cause toxic effects in different systems and hematological indicators affected by ammonia toxicity can be used as important parameters to evaluate ammonia toxicity in aquatic environments.

## Figures and Tables

**Table 1:** Hematological parameters in fish exposed to ammonia.

Exposure Route	Exposure Type	Fish Specie	Ammonia Concentration	Exposure Time	Response Concentration	Response *	Reference
RBC (Billion/mL)							
Sea water	Waterborne exposure	*Takifugu rubripes*	5, 50, 100, 150 mg/L	24, 48, 96 h	100, 150 mg/L	−	Gao et al. [40] 2021
Freshwater	Waterborne exposure	*Cyprinus carpio*	106mg/L	24 h	106 mg/L	−	Hoseini et al. [43] 2019
		*Megalobrama amblycephala*	5, 10, 15, 20 mg/L	9 weeks	20 mg/L	−	Zhang et al. [11] 2019
Ht (%)							
Sea water	Waterborne exposure	*Takifugu rubripes*	5, 50, 100, 150 mg/L	24, 48, 96 h	50, 100, 150 mg/L	−	Gao et al. [40] 2021
		*Piaractus* *mesopotamicus*	1, 2, 3 mg/L	96 h	2, 3 mg/L	+	Edison et al. [57] 2015
Freshwater	Waterborne exposure	*Cyprinus carpio*	106 mg/L	24 h	10 6mg/L	−	Hoseini et al. [43] 2019
		*Megalobrama amblycephala*	5, 10, 15, 20 mg/L	9 weeks	20 mg/L	−	Zhang et al. [11] 2019
Hb (g/L)							
Sea water	Waterborne exposure	*Takifugu rubripes*	5, 50, 100, 150 mg/L	24, 48, 96 h	50, 100, 150 mg/L	−	Gao et al. [40] 2021
		*Piaractus* *mesopotamicus*	1, 2, 3 mg/L	96 h	2, 3 mg/L	−	Edison et al. [57] 2015
Freshwater	Waterborne exposure	*Cyprinus carpio*	106 mg/L	24 h	106 mg/L	−	Hoseini et al. [43] 2019
		*Megalobrama amblycephala*	5, 10, 15, 20 mg/L	9 weeks	20 mg/L	−	Zhang et al. [11] 2019
Glucose (mg/dL)							
Sea water	Waterborne exposure	*Takifugu rubripes*	5, 50, 100, 150 mg/L	24, 48, 96 h	50, 100, 150 mg/L	+	Gao et al. [40] 2021
		*Litopenaeus vannamei*	0.32, 0.44, 0.60 mg/L	6 h, 12 h, 1 day, 2 days	0.32, 0.44, 0.60 mg/L	+	Cui et al. [53] 2017
		*Piaractus* *mesopotamicus*	1, 2, 3 mg/L	96 h	2, 3 mg/L	+	Edison et al. [57] 2015
Freshwater	Waterborne exposure	*Pelteobagrus fulvidraco*	100 mg/L	24, 48, 72 h	100 mg/L	+	Zhao et al. [52] 2021
		*Megalobrama amblycephala*	5, 10, 15, 20 mg/L	9 weeks	20 mg/L	−	Zhang et al. [11] 2019
		*Cyprinus carpio*	0.5 mg/L	24 h	0.5 mg/L	+	Mirghaed et al. [58] 2019
Total protein (g/dL)							
Sea water	Waterborne exposure	*Takifugu rubripes*	5, 50, 100, 150 mg/L	24, 48, 96 h	50, 100, 150 mg/L	−	Gao et al. [40] 2021
		*Epinephelus fuscoguttatus* ♀ *× E. lanceolatus* ♂	1, 2, 4, 8 mg/L	1week, 2 weeks	8 mg/L	−	Kim et al. [48] 2020
Freshwater	Waterborne exposure	*Pelteobagrus fulvidraco*	100 mg/L	24, 48, 72 h	100 mg/L	+	Zhao et al. [52] 2021
	Inject	*Ctenopharynodon idellus*	9 μL	96 h	9 μL	×	Xing et al. [59] 2016
AST (U/L)							
Freshwater	Waterborne exposure	*Pelteobagrus fulvidraco*	100 mg/L	24, 48, 72 h	100 mg/L	+	Zhao et al. [52] 2021
		*Megalobrama amblycephala*	5, 10, 15, 20 mg/L	9 weeks	20 mg/L	+	Zhang et al. [11] 2019
		*Cyprinus carpio*	106 mg/L	24 h	106 mg/L	+	Hoseini et al. [43] 2019
ALT (U/L)							
Sea water	Waterborne exposure	*Takifugu rubripes*	5, 50, 100, 150 mg/L	24, 48, 96 h	50, 100, 150 mg/L	+	Gao et al. [40] 2021
Freshwater	Waterborne exposure	*Pelteobagrus fulvidraco*	100 mg/L	24, 48, 72 h	100 mg/L	+	Zhao et al. [52] 2021
		*Megalobrama amblycephala*	5, 10, 15, 20 mg/L	9 weeks	20 mg/L	×	Zhang et al. [11] 2019
		*Cyprinus carpio*	106 mg/L	24 h	106 mg/L	+	Hoseini et al. [43] 2019
ALP (U/L)							
Freshwater	Waterborne exposure	*Pelteobagrus fulvidraco*	100 mg/L	24, 48, 72 h	100 mg/L	×	Zhao et al. [52] 2021
		*Megalobrama amblycephala*	5, 10, 15, 20 mg/L	9 weeks	20 mg/L	×	Zhang et al. [11] 2019
		*Cyprinus carpio*	106 mg/L	24 h	106 mg/L	+	Hoseini et al. [43] 2019

* +: increase, −: decrease, ×: no effect.

**Table 2:** Antioxidant enzyme responses such as SOD, CAT and GST in fish exposed to ammonia.

Exposure Route	Exposure Type	Fish Specie	Ammonia Concentration	Exposure Periods	Response Concentration	Target Organs	Response *	Reference
SOD (Superoxide dismutase)								
Sea water	Waterborne exposure	*Dicentrarchus labrax*	20 mg/L	12, 48, 84, 180 h	20 mg/L	Blood	×	Sinha et al. [76] 2015
		*Epinephelus fuscoguttatus* ♀ *× E. lanceolatus* ♂	1, 2, 4, 8 mg/L	1week, 2 wk	4, 8 mg/L	Liver, Gill	+	Kim et al. [48] 2020
		*Scophthalmus maximus*	5, 20, 40 mg/L	24, 48, 96 h	20, 40 mg/L	Liver	+	Jia et al. [98] 2020
		*Chlamys farreri*	20 mg/L	1, 12, 24 d	20 mg/L	Blood	+	Wang et al. [85] 2012
		*Takifugu rubripes*	5, 50, 100, 150 mg/L	24 h	50, 100, 150 mg/L	Gill	+	Gao et al. [40] 2021
				48, 96 h	50, 100, 150 mg/L	Gill	−	
Freshwater	Waterborne exposure	*Carassius auratus*	10, 50 mg/L	30 d	10, 50 mg/L	Liver	−	Qi et al. [9] 2017
		*Megalobrama amblycephala*	5, 10, 15, 20 mg/L	9 weeks	20 mg/L	Liver	−	Zhang et al. [11] 2019
		*Cyprinus carpio*	106 mg/L	24 h	106 mg/L	Blood	×	Hoseini et al. [43] 2019
		*Oreochromis niloticus*	5, 10 mg/L	70 days	5, 10 mg/L	Liver, Muscle	+	Hegazi et al. [87] 2010
CAT (Catalase)								
Sea water	Waterborne exposure	*Dicentrarchus labrax*	20 mg/L	12, 48, 84, 180 h	20 mg/L	Blood	+	Sinha et al. [76] 2015
		*Scophthalmus maximus*	5, 20, 40 mg/L	24, 48, 96 h	20, 40 mg/L	Liver	+	Jia et al. [98] 2020
		*Takifugu rubripes*	5, 50, 100, 150 mg/L	24 h	50, 100, 150 mg/L	Gill	+	Gao et al. [40] 2021
				48, 96 h	50, 100, 150 mg/L	Gill	−	
Freshwater	Waterborne exposure	*Carassius auratus*	10, 50 mg/L	30 days	10, 50 mg/L	Liver	×	Qi et al. [9] 2017
		*Megalobrama amblycephala*	5, 10, 15, 20 mg/L	9 weeks	20 mg/L	Liver	−	Zhang et al. [11] 2019
		*Cyprinus carpio*	106 mg/L	24 h	106 mg/L	Blood	−	Hoseini et al. [43] 2019
		*Corbicula fluminea*	10, 25 mg/L	24, 48 h	10 mg/L	Digestive gland	+	Zhang et al. [84] 2020
			10, 25 mg/L	24, 48 h	10 mg/L	Gill	×	
			10, 25 mg/L	24, 48 h	25 mg/L	Digestive gland	−	
			10, 25 mg/L	24, 48 h	25 mg/L	Gill	+	
GST (Glutathione-S-transferase)								
Sea water	Waterborne exposure	*Dicentrarchus labrax*	20 mg/L	12, 48, 84, 180 h	20 mg/L	Blood	+	Sinha et al. [76] 2015
		*Takifugu rubripes*	5, 50, 100, 150 mg/L	24 h	50, 100, 150 mg/L	Gill	+	Gao et al. [40] 2021
				48, 96 h	50, 100, 150 mg/L	Gill	−	
		*Epinephelus fuscoguttatus♀* × *E. lanceolatus* *♂*	1, 2, 4, 8 mg/L	1 week	4, 8 mg/L	Liver, Gill	+	Kim et al. [48] 2020
		*Epinephelus fuscoguttatus♀* × *E. lanceolatus* *♂*	1, 2, 4, 8 mg/L	2 weeks	4, 8 mg/L	Liver, Gill	−	
Freshwater	Waterborne exposure	*Carassius auratus*	10, 50 mg/L	30 days	10, 50 mg/L	Liver	×	Qi et al. [9] 2017
		*Paralichthys orbignyanus*	5, 10 mg/L	70 days	5, 10 mg/L	Liver, Muscle	+	Hoseini et al. [43] 2019
		*Cyprinus carpio* L.	10, 20, 30 mg/L	6, 24, 48 h	30 mg/L	Liver	+	Li et al. [10] 2019
			10, 20, 30 mg/L	6, 24, 48 h	10, 20, 30 mg/L	Gill	+	

* +: increase, −: decrease, ×: no effect.

## Data Availability

Data are contained within the article.
